# The Pyrogallic Acid Treatment of Phagedenæ

**Published:** 1883-02

**Authors:** 


### The Pyrogallic Acid Treatment of Phagedenæ.

The application of pyrogallic acid to phagedenic chancres is followed by such excellent results that we frequently return to the application used by M. Vidal during his service. There were in the ward two cases in which the phagedenæ had attained considerable size, and two or three applications of the pyrogallic acid sufficed to completely arrest the extension of the lesion. Two days later Vidal practiced auto-inoculation in these cases and the result was negative; he has, in fact, been able to demonstrate that the liquid secreted from the wound after the use of the acid loses completely its virulence. These tests can now be made with impunity, because we have in the pyrogallic acid a means of stopping at once the progress of any injuries that might be produced. The application of the pyrogallic acid gives the wound a blackish color, about which there need be no concern. It should also be mentioned that this treatment induces about the base of the sore a slight induration which greatly resembles that observed in the syphilitic chancre, and might easily lead to error if one were not informed of this result. In cases where the wound was rather large and presented numerous irregularities or cavities, necessarily attended with considerable difficulty in applying the remedy to all its parts, instead of applying the salve he ordinarily used, M. Vidal makes application to its surface of a powder composed of one part of pyrogallic acid to four parts of starch. This application is renewed morning and evening, two successive days of the treatment generally being sufficient to completely modify the phagedenic character of the wound. The treatment is then continued by means of the subcarbonate of iron.—*Journal de Med. et de Chirurgie.*

